# Comparison of GnRH agonist and hCG for priming *in vitro* maturation cycles in cancer patients undergoing urgent fertility preservation

**DOI:** 10.1371/journal.pone.0208576

**Published:** 2018-12-06

**Authors:** Hady El Hachem, Charlotte Sonigo, Julie Benard, Marion Presse, Christophe Sifer, Nathalie Sermondade, Michael Grynberg

**Affiliations:** 1 Department of Reproductive Medicine, Ovo Clinic, Montreal, Quebec, Canada; 2 Department of Obstetrics and Gynecology, University of Montreal, Montreal, Quebec, Canada; 3 Department of Reproductive Medicine & Fertility Preservation, Hôpital Jean Verdier, Bondy, France; 4 Inserm U1185, Université Paris-Sud, Le Kremlin Bicêtre, France; 5 University Paris XIII, Paris, France; 6 Department of Cytogenetic and Reproductive Biology, Hôpital Jean Verdier, Bondy, France; 7 Inserm U1133, Université Paris-Diderot, Paris, France; John Hopkins University School of Medicine, UNITED STATES

## Abstract

*In vitro* maturation **(**IVM) of oocytes retrieved at germinal vesicle or Metaphase I stage, followed by vitrification of Metaphase II (MII) oocytes, has recently emerged as an option for urgent fertility preservation (FP). Priming is usually achieved with an injection of hCG, 10,000 IU, 36 hours before retrieval. This study aimed to assess a new method of priming, using GnRH agonists, and compare it to hCG, in cancer patients undergoing urgent FP. From 2009 to 2015, 373 cancer patients underwent MII oocyte cryopreservation after IVM cycles primed either with GnRHa (triptorelin 0.2 mg) (n = 138) or hCG (10,000 IU) (n = 235). Patients’ characteristics were comparable between the two groups. The number of COC retrieved was significantly higher in the GnRHa group (9.1 ± 6.8 versus 7.7 ± 5.5 oocytes, p = 0.04). However, the maturation rates (59 ±25% versus 64 ±26%, p = 0.07, respectively), and the total number of MII oocytes frozen (5.2 ±4.2 versus 4.9 ±4.0, p = 0.6, respectively) were similar between the GnRha and hCG groups. We did not find any difference between GnRHa and hCG priming for IVM. GnRHa priming is more physiological since it stimulates endogenous FSH and LH activity, and is well suited for FP in hormone-sensitive cancers and urgent cases.

## Introduction

Fertility preservation (FP) is nowadays an integral part of the initial management of young cancer patients. It is currently recommended that all patients be counseled regarding the reproductive risks of cancer treatments, and referred to a reproductive specialist to discuss FP before the start of gonadotoxic therapy [1–3]. Among the many FP methods currently available, controlled ovarian hyperstimulation (COH) followed by cryopreservation of oocytes and/or embryos is the most established [1–3]. However, COH might not be always feasible, such as in patients with estrogen-sensitive malignancies, and in urgent cases with timing constraints. For these patients, *in vitro* maturation (IVM) of oocytes has been proposed as an alternative method [4–6]. IVM involves retrieval of immature cumulus-oocyte complexes (COCs) from small antral follicles without previous exogenous gonadotropin administration, followed by *in vitro* maturation in culture media during which oocytes develop from germinal vesicle (GV) or metaphase I (MI) to metaphase II (MII) stage. Oocytes having reached MII stage can then be frozen or fertilized for embryo cryopreservation [5–7].

Hormonal priming, which involves administration of low doses of gonadotropins before COCs retrieval, has been proposed in order to promote oocyte competence *in vivo* and improve outcomes of IVM cycles [8,9]. Various priming protocols have been described, including low doses of FSH [9], a single injection of human chorionic gonadotropin (hCG) [8], or a combination of these two hormonal activities [10]. Many studies have assessed the different approaches, but results have been heterogeneous, mainly because of the variations in the indications, the doses used and the populations analyzed.

Recently, GnRH agonists (GnRHa) have been used for induction of final oocyte maturation in COH with GnRH antagonist cycles. GnRHa trigger allows retrieving matured oocytes in high responders to ovarian stimulation, while significantly reducing the risk of ovarian hyperstimulation syndrome (OHSS) by decreasing the duration of LH stimulation of the luteinized granulosa/theca cells [11]. Studies have now confirmed that the number of oocytes retrieved, as well as the maturation and fertilization rates achieved with a GnRHa trigger are comparable to those reported with a conventional hCG trigger in COH cycles [12–14].

Based on the physiological rationale and the abundant evidence obtained from COH cycles supporting the use of GnRHa trigger, we recently described a novel priming protocol with GnRHa in IVM cycles performed for FP [15]. Following encouraging results, we decided to continue the strategy of GnRHa priming and to compare its effectiveness with the classical hCG priming in women undergoing IVM for FP before gonadotoxic treatment. The main outcome of the present investigation was the number of MII oocytes available for cryopreservation.

## Materials and methods

### Subjects

From January 2009 to December 2015, cancer patients, 18–40 years of age, referred to our center for urgent FP using IVM, were studied. All met the following inclusion criteria: (i) presence of two ovaries; (ii) adequate visualization of ovaries at transvaginal ultrasound scans; (iii) total number of small antral follicles (3–12 mm in diameter) >8. We included patients in the follicular as well as the luteal phase at the time of retrieval. Patients with a previous history of chemotherapy or diagnosed with polycystic ovarian syndrome (PCOS) according to the Rotterdam criteria [16] were excluded from the present investigation. Single patients were offered oocyte cryopreservation but if they were in relationship they had the possibility of choosing oocyte or embryo freezing.

Before oncofertility counseling, each woman underwent a blood sampling by venipuncture for measurement of serum AMH and progesterone levels, and a transvaginal ultrasound scan for antral follicle assessment. Ultrasound scans were performed by 2 operators, who were blinded to the results of hormone assays. All follicles measuring 3 to 22 mm in mean diameter (mean of two orthogonal diameters) were considered. To optimize the reliability of ovarian follicular assessment, the ultrasound scanner used was equipped with a tissue harmonic imaging system [17], which allowed improved image resolution and adequate recognition of follicular borders. Follicles measuring >12 mm were considered as dominant and not included in the antral follicle count (AFC). Luteal phase was defined by the presence of a corpus luteum and a serum progesterone level > 3 ng/mL.

### Study groups and IVM technique

None of the patients received any ovarian stimulation with gonadotropins, and all received priming with LH activity 36 hours before retrieval. From January 2009 to April 2012 IVM cycles were primed with urinary hCG (Gonadotrophine Chorionique Endo, MSD, Courbevoie, France, 10,000 IU IM; hCG group). From May 2012 onwards, LH activity was systematically provided using GnRHa (triptorelin, Ipsen Pharma, Boulogne-Billancourt, France, 0.2 mg SC; GnRHa group).

Oocyte retrieval was performed using a technique described elsewhere [18]. Follicular fluid was analyzed in Nucleon culture dishes (Nunc A/S, Denmark), where COCs were isolated and washed with a culture medium, Universal IVF Medium (Origio, Denmark). COCs were then placed into a culture dish (Becton Dickinson, USA) containing 1 mL of culture medium IVM (Medi Cult, Danemark) enriched with 20% inactivated maternal serum 0.75 UI/mL of, FSH and 0.75 UI/mL of LH Menopur (Ferring, Germany) [19]. COCs were then incubated at 37^o^ C in a 5% CO_2_/20% O_2_/N_2_ gas mixture. After 24 hours of culture, all COCs were denuded with a hyaluronidase solution (SynVitro Hyadase, Origio, Denmark) and nuclear oocyte maturation was assessed. Oocytes having failed to mature after 24 hours were kept for an additional 24 hours of culture. After 48 hours, oocytes that reached MII stage were frozen, while immature eggs were discarded. Finally, depending on the patient’s choice, mature MII oocytes were frozen on the same day or fertilized by ICSI and zygotes were frozen at day 1 of embryo development.

### Oocyte retrieval rate (ORR)

To objectively assess the efficiency of immature oocyte retrieval in GnRHa and hCG groups, we decided to analyze the oocyte retrieval rate (ORR), calculated as the ratio of the number of COCs recovered x100/AFC (3–12 mm in diameter).

### Oocyte / Zygote cryopreservation

Since vitrification was unauthorized in France before July 2011, all mature oocytes obtained before this date were cryopreserved using the slow-freezing method [20], according to the manufacturer recommendations (OocyteFreeze, Origio, Denmark). From July 2011 onwards, oocytes or embryos were vitrified [21]. Until June 2013, vitrification was performed using the closed Rapid-i vitrification system (Vitrolife, France) and Blast-freeze media (Vitrolife) as recommended by Vitrolife for oocyte or zygote cryopreservation. The procedure was performed according to the manufacturer’s instructions. Since July 2013, vitrification of oocytes and zygotes were performed using Kitazato Vitrification Kit (Kitazato BioPharma Co., Ltd., Japan) or Embryo Vitrification Freeze Kit (Irvine Scientific, USA), respectively, according to manufacturers’ instructions.

### Statistical analysis

The measures of central tendency and variability used were the mean ± standard deviation (SD). Differences between continuous variables from the GnRHa and hCG groups were evaluated with Student’s t test with Welsh correction. Categorical variables in the two groups were compared using the two-sided Pearson x2 test. A *p* value <0.05 was considered statistically significant.

The present investigation was approved by the “Jean Verdier Hospital review board”, and ethical clearance was obtained from the “Human ethical clearance committee (Comité Local d’Ethique pour la Recherché Clinique des HUPSSD Avicenne- Jean Verdier -René Muret (CLEA) (CLEA-2015-020)”. All patients signed an informed consent before being included. All IVM protocols were performed in accordance with the institutional guidelines and national regulations.

## Results

Overall, 373 patients met the inclusion criteria. The main indication for FP with IVM was breast cancer requiring urgent neoadjuvant chemotherapy or advanced stage with contraindication for COH (n = 329). Other indications were hematological malignancies (n = 15), and various malignant diseases requiring urgent chemotherapy (n = 29).

138 women received GnRHa priming (GnRHa group; n = 138) and 235 women received hCG priming (hCG group; n = 235). Baseline characteristics of patients included in both groups are shown in Table 1. There were no significant difference between the GnRHa and hCG groups in terms of age (31.7 ± 4.4 versus 32.2 ± 4.9 years, *p = 0*.*4*, respectively), body mass index (BMI) (23.2 ± 4.3 versus 22.8 ± 3.9 Kg/m^2^, *p = 0*.*4*, respectively) and ovarian reserve markers (AFC: 18.8 ± 7.6 versus 18.6 ± 8.0 follicles, *p = 0*.*8*; serum AMH levels: 3.7 ± 2.4 versus 3.6 ± 2.7 ng/mL, *p = 0*.*9*, respectively).

**Table 1:** Patients’ characteristics.

Variable	GnRHa *n = 138*	hCG *n = 235*	p
Age (years) ^a^	31.7 ± 4.4	32.2 ± 4.9	*0*.*4*
BMI (Kg/m2)^a^	23.2 ± 4.3	22.8 ± 3.9	*0*.*4*
AFC ^a^	18.8 ± 7.6	18.6 ± 8.0	*0*.*8*
AMH (ng/mL) ^a^	3.7 ± 2.4	3.6 ±2.7	*0*.*9*

^a^:Mean ± SD

IVM outcomes are reported in Table 2. The number of COCs retrieved (9.1 ± 6.8 versus 7.7 ± 5.5 oocytes, *p = 0*.*04*) as well as the ORR (52 ± 31% versus 42 ± 25%, *p = 0*.*005*) were significantly higher in the GnRHa group compared to the hCG group. There was a non-significant trend towards a reduced oocyte maturation rate after 48 hours with GnRHa priming (59 ± 25% versus 64 ± 26%, *p = 0*.*07*). Finally, the total number of IVM oocytes available for cryopreservation was comparable between the GnRHa and hCG groups (5.2 ± 4.2 versus 4.9 ± 4.0, *p = 0*.*6*, respectively). 8 MII oocytes were retrieved from 8 patients in the GnRHa group compared to 19 MII oocytes in 19 patients in the GnRHa group, and more oocytes were mature after 24 hours in the hCG group (87 ± 22% vs 92 ± 17%, p = 0.03).

**Table 2:** IVM results according to the type of priming.

Variable		GnRHa *n = 138*	hCG *n = 235*	p
Cycle phase	Follicular phase (%)	51% *(n = 70)*	59% *(n = 138)*	0.2
	Luteal phase (%)	49% *(n = 68)*	41% *(n = 97)*	
No oocytes retrieved ^a^		9.1 ± 6.8	7.7 ± 5.5	0.04
ORR (%) ^b^		52 ± 31	42 ± 25	0.005
Maturation rate after 48hrs (%) ^c^		59 ± 25	64 ± 26	0.07
No total of oocytes vitrified after IVM ^a^		5.2 ±4.2	4.9 ± 4.0	0.6

^a^ Mean ± SD

^b^ OOR: Oocyte Output Rate: number of COCs recovered x100/antral follicle count (AFC, 3–12 mm in diameter)

^c^ Number of MII oocytes x100/No of COCs recovered

The phase of the cycle during which oocyte retrieval was performed was also comparable between the GnRHa and hCG groups (follicular phase: 51% (70) versus 59% (138); luteal phase: 49% (68) versus 41% (97), *p = 0*.*2*, respectively), as well as the number of IVM cycles with presence of a dominant follicle (8.7% versus 14.0%, p = *0*.*12*). When comparing cycles performed in the follicular phase, the number of COCs retrieved (8.6 ± 6.2 vs 7.2 ± 5.3, p = 0.09) was comparable between the GnRHa and hCG group, but the ORR was significantly higher in the GnRHa group (51 ± 33%, vs 40 ± 25%, p = 0.01). However, with similar maturation rates after 48 hours (60 ± 25% vs 67 ± 28%, p = 0.1), the total number of mature oocytes (5.2 ± 4.4 vs 4.7 ± 3.7, p = 0.39) was also comparable between the GnRHa and hCG group. For cycles performed in the luteal phase, all outcomes were comparable between the GnRHa and hCG groups: number of oocytes retrieved (9.7 ± 7.3 vs 8.4 ± 5.6, p = 0.19), ORR (51 ± 28% vs 44 ± 25%, p = 0.09), maturation rate after 48 hours (58 ± 24% vs 61± 24%, p = 0.43), and number of mature oocytes (5.2 ± 3.9 vs 5.2 ± 4.3, p > 0.9).

Finally, when including only cycles without a dominant follicle (130 in the GnRHa group compared to 213 in the hGC group), the total number of mature oocytes was also comparable between the two groups (4.9 ± 4.0 vs 5.0 ± 4.1, p = 0.9), even though the level of significance varied for the number of COC’s retrieved (9.0 ± 6.7 vs 7.7 ± 5.5, p = 0.07), the ORR (50 ± 31 vs 42 ± 25, p = 0.02), and the maturation rate (58 ± 22 vs 65 ± 26, p = 0.04) between the GnRHa and the hCG group, respectively.

Most women had oocyte cryopreservation (n = 330), and only 43 opted for embryo freezing. We were not able to compare the fertilization rate and the embryo morphology between the two priming groups since only six patients opted for embryo cryopreservation in the GnRHa group compared to 37 in the hCG group.

## Discussion

To the best of our knowledge, this is the first study reporting on GnRHa priming for IVM. We compared this new priming method to the widely used hCG priming in patients undergoing IVM for urgent FP, and found a significantly better oocyte retrieval rate with GnRH agonists. However, the final number of mature MII oocytes available for cryopreservation was comparable between the two methods.

IVM was initially proposed as an alternative to COH in a bid to reduce the risks, costs and side effects and was first adopted in women with PCOS at high risk of OHSS. However, despite the significant progress since the first live birth was reported in 1991 [22], implantation and live birth rates (LBRs) have remained lower than those of conventional IVF [23,24]. In an effort to improve IVM success rates, different hormonal primings were proposed, aiming to promote oocyte competence *in vivo*. Thus, administration of low doses of FSH (37.5–150 IU/day for 3 to 6 days) was reported to improve IVM outcomes by increasing the number and diameter of antral follicles, making them more accessible and easier to puncture [9,25]. In addition, hCG has also been used for priming, and studies have shown that it promotes *in vivo* meiotic resumption, GV breakdown and oocyte maturation, as well as cumulus cells expansion, which facilitates detachment and expulsion of the COC mass from the follicle during the aspiration and thus makes oocyte collection easier [26–28]. In women with PCOS, priming with low doses of FSH [9,29,30], or a single dose of hCG (10,000 IU administered 36 h before oocyte retrieval) [8,31–34] improves oocyte maturation and pregnancy rates, while a combination of both failed to show a significant improvement in overall outcomes [35]. In normo-ovulatory women, the impact of priming with FSH or hCG has been different according to studies [9,27,36]. In a prospective randomized study, Fadini *et al*. compared four different priming approaches in normo-ovulatory women: no priming, hCG (10,000 IU), FSH (150 IU/d for 3 days from day 3), and FSH/hCG, and found that, even though the immature oocyte yield was comparable between the different protocols, the FSH/hCG priming significantly improved oocyte maturation both *in vivo* and *in vitro*, as well as clinical pregnancy rates. Moreover, FSH priming or hCG priming alone had no significant effect on the clinical outcome [10]. Combined FSH/hCG priming is now considered the preferred approach for IVM cycles in normo-ovulatory woman.

GnRH agonists have been successfully used to trigger final oocyte maturation in IVF cycles since the late 1980s and early 1990s [37–40]. However, for years, their use was mainly reserved for pituitary down-regulation during ovarian stimulation requiring hCG triggering before oocyte retrieval. The widespread use of GnRH antagonists in IVF cycles since 2000 has revived the interest in the use of GnRHa to trigger final oocyte maturation [41], particularly as a preventive approach of OHSS [11,14,42,43]. It is now widely accepted that oocyte maturation rates with GnRHa trigger are comparable to those achieved with hCG trigger in antagonist cycles [14,44–48] and some studies have even shown a higher proportion of mature oocytes with GnRHa [12,13,49]. A single bolus of GnRHa is considered to be more physiologic than conventional hCG trigger, since it leads to an endogenous LH and FSH surge, which mimics the natural mid-cycle surge of gonadotropins. Even though the physiologic role of the FSH surge is not fully elucidated, some studies have suggested that it might have an effect on resumption of meiosis and oocyte maturation, expansion and dispersion of the COC [50,51], and release of proteolytic enzymes involved in ovulation [52,53]. Moreover, the greater oocyte maturity reported with GnRHa could be due to the more rapid increase in serum LH after agonist trigger compared to the rise of serum hCG level after a 10,000 IU injection of hCG [54], to the simultaneous FSH surge, or a combination of both.

Based on these findings, we sought to determine whether GnRH agonists could be used for priming IVM cycles in normo-ovulatory women. We chose a specific population of women undergoing urgent FP before gonadotoxic treatment since these patients would benefit the most from the physiological double FSH and LH surge elicited by a single GnRHa injection. Indeed, IVM is considered a possible alternative to COH for FP in cases of hormone sensitive cancers such as breast cancer (BC) where conventional COH could be problematic, and in cases of urgent FP, where the window for treatment before the start of gonadotoxic therapy is dramatically reduced. Many studies have now shown that IVM is an effective method of FP for these patients. Indeed, Shalom-Paz *et al*, reported an average of 11.4 immature oocytes retrieved and 7.9 MII oocytes cryopreserved in 66 BC patients undergoing FP with IVM [55]. In addition, we recently showed, in 248 BC patients, that the retrieval of immature oocytes from small antral follicles as well as the IVM rates remain similar whatever the period of the menstrual cycle [18], confirming the results described by Maman *et al*. in a small series [56]. All these studies used conventional hCG priming before oocyte retrieval. In the current study, we found the number of immature oocytes retrieved to be significantly higher following GnRHa priming compared to hCG. We also compared the ORR, which is more accurate to evaluate the efficiency of the retrieval procedure, since it takes into account the number of small antral follicles, as opposed to the absolute number of immature oocytes retrieved. The ORR was also significantly higher following GnRHa priming. This finding could be explained by the intrinsic FSH activity stimulated by the GnRHa, which is likely to enhance follicular development, making follicles more visible on ultrasound and easier to puncture. Indeed, this is one of the arguments in favor of the conventional 3-day FSH priming used in IVM cycles in normo-ovulatory women [10,57]. However, such priming is not always feasible, in particular when the FP procedure should be performed in emergency. We found the oocyte maturation rates after 48 hours to be comparable between the two groups, with a non-significant trend towards better maturation with hCG, but both were similar to maturation rates reported in earlier studies [10,18,56]. The added FSH surge therefore does not seem to improve maturation rates, as has been suggested by studies from COH [49–51]. One possible explanation is that the positive impact of the physiological FSH surge on oocyte maturation might only occur in bigger and more mature follicles already exposed to several days of FSH. The total number of MII oocytes cryopreserved was comparable between the two priming protocols, and in accordance with numbers reported with IVM for FP in cancer patients [18,55,56], confirming that GnRHa priming is equally effective as hCG priming for FP.

The mean number of MII oocytes cryopreserved with both priming methods in this study is acceptable and further validates the role of IVM as an effective FP method. However, it is worth noting that the developmental potential of oocytes cryopreserved following IVM remains ill established, with few live births reported in non-cancer patients [58,59], and none in a cancer patient. Moreover, implantation and pregnancy rates following IVM in non-cancer patients remain lower than those reported with COH and *in vivo* matured oocytes [24]. This could be the result of higher aneuploidy rates in embryos obtained from IVM oocytes [60,61], but also other factors, such as the underlying cause of infertility, since most IVM outcomes were reported in patients with PCOS who might have an inherent alteration of their oocytes [62]. Suboptimal endometrial priming and receptivity in IVM cycles has also been identified as a limiting factor, and recent data suggest that a freeze-all strategy and frozen embryo transfer following adequate endometrial preparation significantly improves implantation and pregnancy rates [63], which could be applied for cancer patients undergoing FP with IVM. However, it is conceivable that the more physiologic effect of GnRH agonists priming when compared to hCG, may influence the competence of *in vitro* matured oocytes.

The main limitation of our study is the lack of prospective randomization. However, the two groups were statistically similar in terms of age, body mass index, markers of ovarian reserve and the phase of the cycle during which oocyte retrieval was performed. Moreover, we were not able to compare the fertilization rate and the embryo morphology between the two priming methods since the vast majority of patients opted for oocyte cryopreservation. These parameters, as well as future implantation and pregnancy rates, would allow us to adequately evaluate the efficiency of GnRHa priming.

## Conclusion

The present investigation describes a new method of priming in IVM cycles for FP using GnRH agonists. This approach is equally effective as hCG priming, but is more physiological and is more suited for FP in hormone-sensitive cancers and urgent cases. Randomized control trials are needed to objectively assess the need for priming in normo-ovulatory cancer patients, and the best way to provide it if required.
